# Oral health status of a population with multiple sclerosis

**DOI:** 10.4317/medoral.17340

**Published:** 2011-12-06

**Authors:** Eva Santa Eulalia-Troisfontaines, Eva M. Martínez-Pérez, Mónica Miegimolle-Herrero, Paloma Planells-del-Pozo

**Affiliations:** 1 Honorary associate professor, Universidad Complutense de Madrid; 2 Associate professor, Universidad Complutense de Madrid; 3 Assistant professor, Universidad Europea de Madrid; 4 Titular professor of Pediatric Dentistry, Universidad Complutense de Madrid

## Abstract

Objective: To determine the oral treatment needs of a sample of patients diagnosed with multiple sclerosis in the Community of Madrid (Spain). Patients and methods: A cross-sectional epidemiological study was carried out with a sample of 64 patients who were aged 25 to 77 years. They were distributed into homogeneous age groups: < 46 years, 46-54 years and > 54 years. In order to evaluate the oral health status and treatment requirements, the parameters and guidelines of the WHO were used. Results: The prevalence of caries was 100%, or very close in all three groups. As age increased, the morbidity rate decreased, but the mortality rate increased considerably. On analyzing gingival health, 65% of patients had calculus, 5% bleeding and 30% were healthy. Conclusions: The DMFT index found provided data that was, in general, very similar to that of the general population in Spain. However, the gingival health status found demonstrated that the population of multiple sclerosis patients requires specific assistance.

** Key words:** Multiple sclerosis, oral health, dentures.

## Introduction

Multiple sclerosis is a chronic autoimmune, inflammatory, and demyelinating disease. It is characterized by the appearance of demyelinating plaques that reduce the efficiency of nerve impulse conduction, leading to motor and sensory symptoms in addition to disturbing the cognitive functions of the individual. It has no cure (1-3).

A part from the usual signs and symptoms associated with multiple sclerosis, the patients who suffer this disease tend have oral pathologies that can diminish or disrupt their quality of life. The fact that there is very little in the literature on the most common oral pathologies of this population, led to this study being undertaken in order to answer the following question: do patients suffering from multiple sclerosis require, given their oral health status, specific protocols to be set-up regarding prevention and care?

## Objective

The aim of this study was to investigate the oral treatment needs of a sample of patients diagnosed with multiple sclerosis in the Community of Madrid.

## Patient and Methods

A cross-sectional epidemiological study was carried out with a target patient population diagnosed with multiple sclerosis who had been admitted into the Multiple Sclerosis Center Alicia Koplowich in the Community of Madrid.

Inclusion criteria:

- Patients diagnosed with multiple sclerosis who had been admitted to the Multiple Sclerosis Center Alicia Koplowich in the Autonomous Community of Madrid.

- Patients who had signed a written consent form, before the study had been carried out. 

Exclusion criteria:

- Patients with a general pathology that made a proper intraoral examination impossible.

- Those patients who did not have a signed consent form before the study.

The examination was carried out by a single operator, a dentist who had specialized in Orodental Preventive programs for individuals and the community (UCM). It was carried out in a reclining chair or in the patient’s own chair in cases of motor disability. 

In order to carry out the examination, sterile examination kits in individual bags were used, as well as paper towels, white light lamps, cotton gauze and swabs and individual examination cards by the WHO. 

The sociodemographic category variables used were age and sex. The health variables studied were DMFT index, indexes for dental morbidity and tooth mortality, caries prevalence, significant caries index and restoration index. 

In order to evaluate dental and periodontal status as well as treatment needs, the parameters and guidelines of the WHO were used. 

The data was collected and then it was treated and classified using the Microsoft Access 2003 and Microsoft Excel 2003 database software. The grouping together, analysis and presentation of estimates was carried out using the SPSS program, version 16. For the descriptive study, the quantitative variables were described such as arithmetic mean +/- standard deviation of the mean and age and sex distribution. 

## Results

The sample chosen for this study was made up of 64 patients with multiple sclerosis whose ages ranged from 25 to 77 years. In accordance with the Law 15/1999, of 13 December regarding Personal Data Protection, four patients of the sample total did not give their consent for the inclusion of part or all of their personal data, such as their date of birth. With regard to age analysis the sample was, as a result, made up of 60 patients.

In order to try to look for behavior patterns in the age variable that were inferable to the population, it was distributed into age groups. Given that the criteria followed specified that the sample sizes of the groups had to be balanced, the patients were divided into three groups: patients under the age of 46, patients aged 46 to 54 years inclusive, and patients over the age of 54. The sample distribution according to sex was 43 females and 21 males. 

 -Analysis of caries as a disease.

 Prevalence of active caries.

For a Confidence Level of more that 95%, significant differences were found between each of the age groups analyzed, and a negative linear relationship was observed between active caries and age increase (age groups). In other words, the prevalence of active caries decreased significantly as age increased. This could be due, as will be seen later on, to the older age group having a higher number of missing teeth due to caries ( Table 1). 

 DMFT index according to age.

In order to ascertain if there was a possible relationship between the age variable (independent variable) and the quantitative variable DMFT (dependent variable), a variance analysis was carried out (one factor ANOVA), with the factor being the ordinal variable age groups.  Table 2, shows that there was no overlapping of the confidence interval between the two higher age groups. This confirmed the existence of significant differences between these two groups as the confidence level was over 95%, while in the other groups this was not the case.

Given that in the ANOVA summary, the critical level was lower than 0.05 (0.007), the null hypothesis of equal means for the DMFT index of each of the groups had to be rejected. In other words, the existence had to be considered of a statistically significant relationship (p<0,05) between age (for the established age groups) and DMFT index.

In order to ascertain which was the group, or groups, with the significant differences, a post hoc test was carried out. The test carried out was the Tukey HSD test for multiple comparisons. It was ascertained that, with a confidence level of over 95%, there were significant differences in the DMFT values between the 46-54 and over 54 age groups. 

 DMFT index according to sex.

As in the previous analysis, which was to ascertain the existence of a relationship between DMFT index -the mean- and sex, a Student’s T-test was carried out. The result indicated that with a confidence level of over 95%, the null hypothesis of equal means could not be rejected ( Table 3). It can therefore be concluded that there was no statistically significant relationship between sex and DMFT index.

**Table 1:**
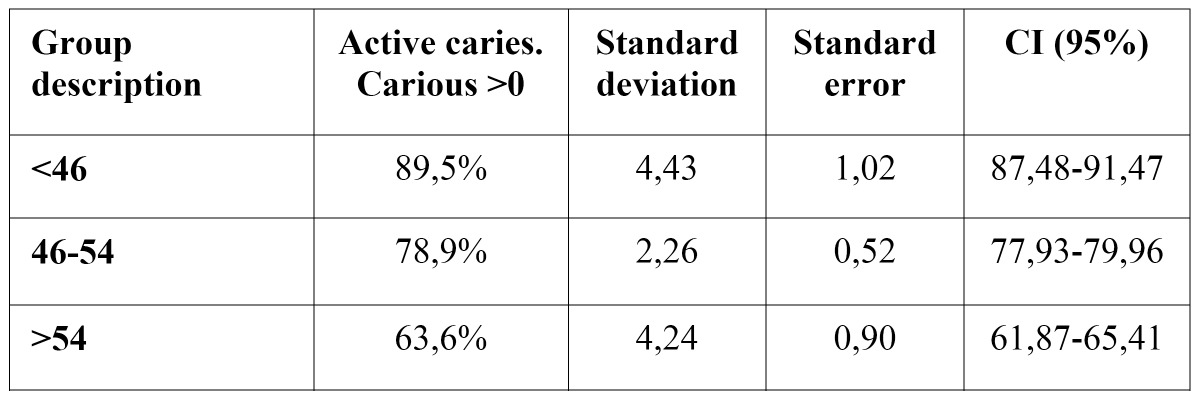
Prevalence of active caries of the age groups

**Table 2:**
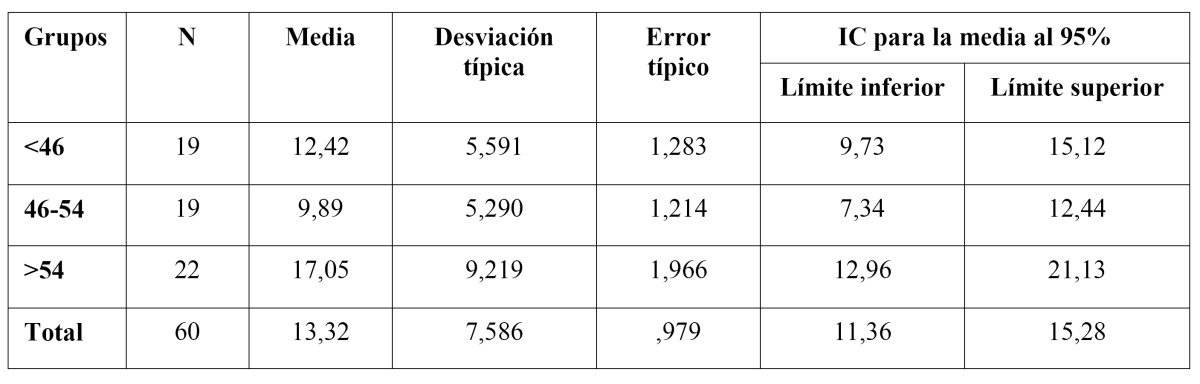
DMFT, means and confidence intervals of age groups.

**Table 3:**
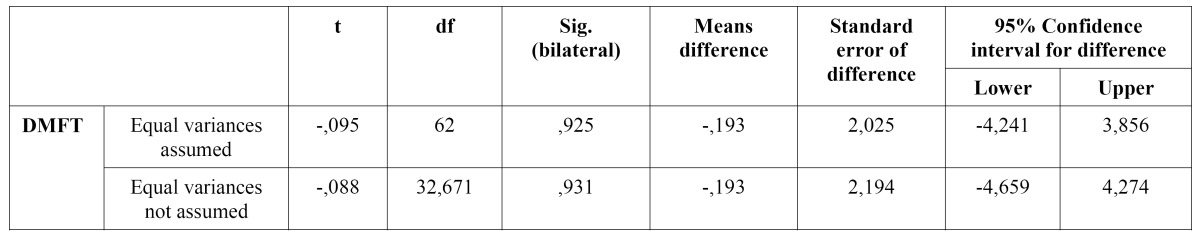
Student’s T-test for comparing means of DMFT and sex (Independent samples).

**Table 4:**
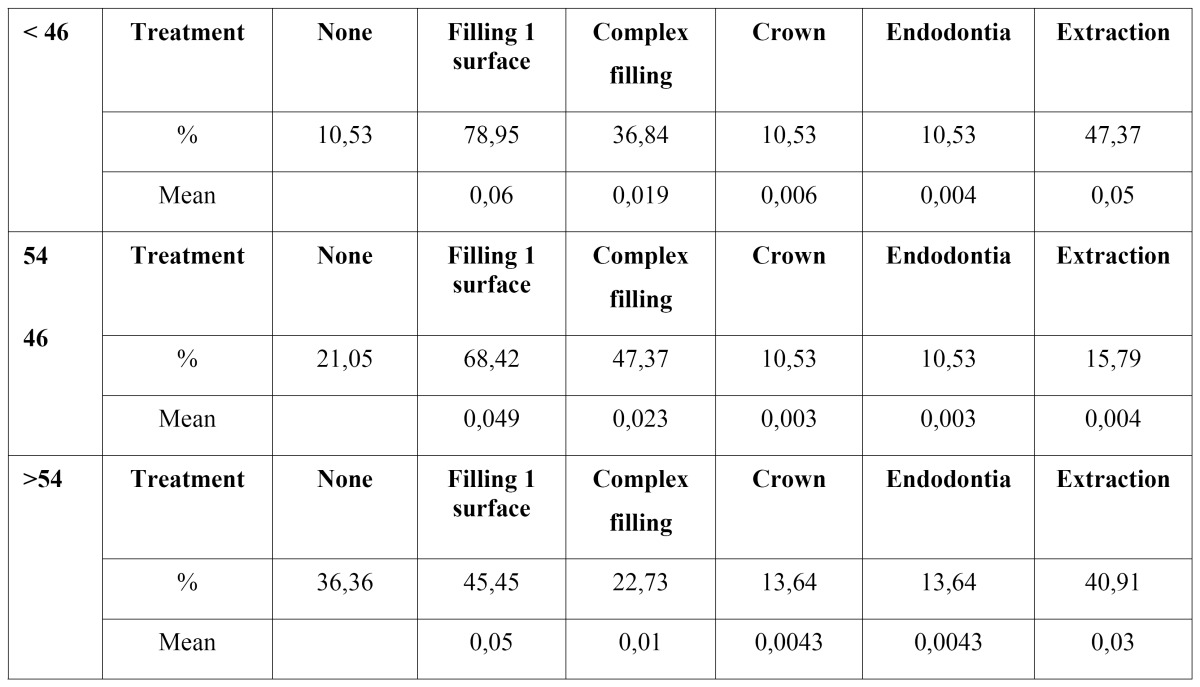
Need for dental restoration or extraction.

 Other caries rates.

**Figure 1:**
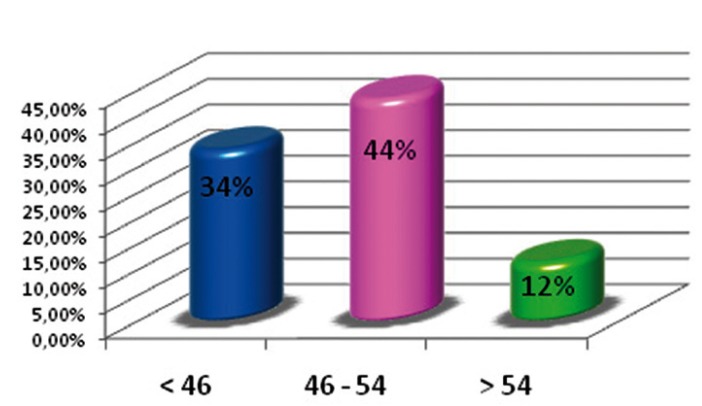
Index of restorations.

**Figure 2:**
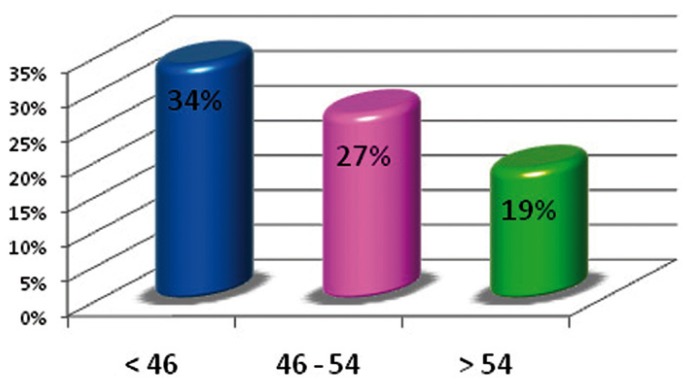
Index for dental morbidity.

**Figure 3:**
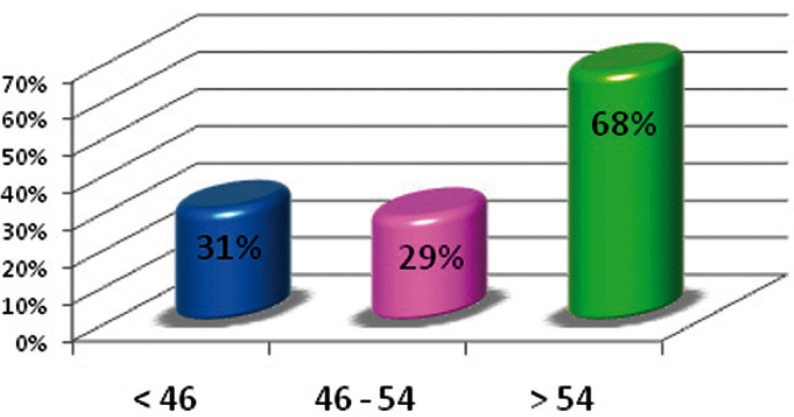
Index for dental mortality.

Figure 1 shows that the oldest age group had a lower restoration index than the rest of the groups, given that the number of filled teeth decreased when compared to the lower age groups. The rate of dental morbidity had a negative lineal relationship because, as age increased, the number for active caries decreased (Fig. 2). The rate of tooth mortality was over 68% in the older age group. This was because the number of missing teeth due to caries in this group was much larger than in the other two groups (Fig. 3). 

 -Necessary restoration treatment.

 Table 4 shows the results of the sample’s treatment requirements. Five different treatment requirements were differentiated: one-surface fillings, two or more surfaces, root canals, crowns and extractions. As has been seen in the over 54 age group, the need for restoration treatment decreased because of the large number of missing teeth that had accumulated in this group. 

 -Gingival health analysis.

On studying gingival health status, it was observed that 30% of patients in the sample had good gingival health, that 5% had bleeding and 65% of the sample had calculus.

 -Analysis of the use of and need for dentures.

Of the patient total, 85% did not wear dentures, 5% had full dentures and 10% wore some other type of removable dentures. With regard to the need for dentures, 87% of the sample total did not need dentures, 1% needed to repair the dentures they wore, and 11% required partial removable dentures.

## Discussion

This study was carried out in collaboration with the Multiple Sclerosis Center Alicia Koplowitz in the Community of Madrid, following their request, in order to contribute to a greater awareness of the dental needs of the patients with multiple sclerosis who had been admitted to the center. The director of the Center and of the Universidad Complutense de Madrid felt that this was of great interest given the lack of literature and research in this field. 

The methodology in this work followed the guidelines of the WHO, as did the studies by Symons et al. (4) and Kovac et al. (5).

The group of patients with multiple sclerosis studied by Kovac et al. (5) had a DMFT index of 12.58. The data were compared with a control group of individuals who did not have the disease, and it was found that there were no significant differences in DMFT index between both groups. 

Symons et al. (4), who divided their group into age groups, studied the DMFT index of a group of 11 patients who were aged 45 to 54 years, and a DMFT mean was found of 23.54. When this was compared with a group from the general population in their country, significant differences were not found in DMFT index. McGrother et al. (6) found a DMFT index of 19.46 in the sample they analyzed, and significant differences were found regarding the control group of healthy individuals. 

In order to evaluate gingival health status, in the present study a scale of three values was used that were defined as: 0= healthy, 1= bleeding and 2= calculus, according to the WHO scale. Taking into account the highest value for each patient, 30% of the sample had good gingival health, 5% had gingival bleeding and 65% had calculus. 

Symons et al. (4) used the community periodontal index (CPITN) and McGrother et al. (5) used their own scale with three values defined as “good”, “average” and “poor,” but what each value refers to is not clarified. The results reported by Symons et al. (4) regarding gingival health were: 36% of their patients were healthy, 15% had bleeding on probing and 32% had calculus and/or probing depths of more than 4 mm.

When the data from our sample is extrapolated and compared with that of the general population, it can seen that these are similar to the figures obtained in the last survey on oral health carried out in Spain in 2005. Given that the age groups into which the sample was divided and the groups in the oral health survey are not the same, in this present study the different parameter values of the investigation for the 35 to 44 and 67 to 74 age groups were used for the sake of comparison. In the Spanish oral health survey the DMFT index for the 35 to 44 age group was 9.61, and for the 67 to 74 age group it was 16.79. 

Bearing in mind that the DMFT indexes in our sample for the lower and higher age groups was 12.42 and 17.05 respectively, it could be said that the DMFT index seems slightly higher in our sample than in the Spanish oral health survey. However, the values for the significant caries index (SiC) are very similar in both studies. The percentage of DMFT is also around 100% for the Spanish population in general. At this point it should be remembered that both Symons et at. (4) and Kovac el al. (5) compared the DMFT scores of a sample of patients with multiple sclerosis with control groups, and that significant differences were not found between the two groups. 

The sample in this present study appears to have a worse gingival health status than the sample from the oral health survey in Spain carried out in 2005. Calculus was present in 68% of the patients belonging to the first and third age groups in our study, and in the Spanish oral health survey (2005) there was calculus in 47% and 38.5% of the patients in the age groups previously mentioned. However Symons et al. (4) did not find significant differences for CPITN between the group of patients with multiple sclerosis and the control group, although it should be taken into account that the sample in the periodontal study was based on 11 patients. 

This marked difference regarding gingival health status could be explained by the fact that patients with multiple sclerosis find brushing their teeth correctly difficult. This should be kept in mind by the auxiliary staff looking after these patients on a daily basis, especially with regard to those patients with a greater physical disability. 

The therapeutic dental requirements of these patients in our patient sample were mostly simple fillings followed by extractions and complex fillings. 

One of the unprecedented objectives in this work included denture use in the sample studied. This was not reflected in previous investigations on sample populations of multiple sclerosis patients. The results of the present study with regard to the use of dentures were the following: 97% of patients in the under 46 group did not use dentures, and in the over 54 group this figure was 68%. The oral health survey carried out in Spain in 2005 concludes that 83% of the patients aged 35 to 44 years did not use dentures, nor did 36% of the patients who were aged 67 to 74 years. 

This information leads us to reflect on why multiple sclerosis patients use dentures less than the general population. The answer could lie in the very existence of an incapacity, which leads multiple sclerosis patients to be treated differently to healthy patients. 

Given the information found in our study and that in other investigations on multiple sclerosis patients, it can be deduced that these patients do not have a pathology that differs from the general population. However, given that there is a clear reduction in quality of life for those with an oral pathology, individualized and specific preventative programs that are aimed at the needs and degree of disability of each patient should be considered. At the same time it should be kept in mind that maintaining oral hygiene is for these patients a great challenge given their motor dificulties. The fact that many of these patients take medication should also be taken into account as this could have adverse effects on orodental health. 

The lack of studies on the oral health of special needs patients came to light when carrying out this investigation. Improving the quality of life of the general population should include that of every individual, and the challenges that caring for patients with special needs should be faced, so that research in this sense is encouraged in the future.
